# Development of Methodology to Investigate the Surface SMALPome of Mammalian Cells

**DOI:** 10.3389/fmolb.2021.780033

**Published:** 2021-11-18

**Authors:** Kerrie A. Morrison, Kate J. Heesom, Karen J. Edler, James Doutch, Gareth J. Price, Francoise Koumanov, Paul Whitley

**Affiliations:** ^1^ Department of Biology and Biochemistry, University of Bath, Bath, United Kingdom; ^2^ Department of Chemistry, University of Bath, Bath, United Kingdom; ^3^ Centre for Sustainable Circular Technologies, University of Bath, Bath, United Kingdom; ^4^ University of Bristol, Proteomics Facility, Bristol, United Kingdom; ^5^ Rutherford Appleton Laboratory, ISIS Pulsed Neutron and Muon Source, Harwell Oxford, United Kingdom; ^6^ Department of Chemistry, Khalifa University, Abu Dhabi, United Arab Emirates; ^7^ Department for Health, University of Bath, Bath, United Kingdom

**Keywords:** styrene maleic acid, SMA, mass spectrometry proteomics, SMALP, SMALPome

## Abstract

Extraction of membrane proteins from biological membranes has traditionally involved detergents. In the past decade, a new technique has been developed, which uses styrene maleic acid (SMA) copolymers to extract membrane proteins into nanodiscs without the requirement of detergents. SMA nanodiscs are compatible with analytical techniques, such as small-angle scattering, NMR spectroscopy, and DLS, and are therefore an attractive medium for membrane protein characterization. While mass spectrometry has also been reported as a technique compatible with copolymer extraction, most studies have focused on lipidomics, which involves solvent extraction of lipids from nanodiscs prior to mass-spectrometry analysis. In this study, mass spectrometry proteomics was used to investigate whether there are qualitative or quantitative differences in the mammalian plasma membrane proteins extracted with SMA compared to a detergent control. For this, cell surface proteins of 3T3L1 fibroblasts were biotinylated and extracted using either SMA or detergent. Following affinity pull-down of biotinylated proteins with NeutrAvidin beads, samples were analyzed by nanoLC-MS. Here, we report for the first time, a global proteomics protocol for detection of a mammalian cell “SMALPome”, membrane proteins incorporated into SMA nanodiscs. Removal of SMA from samples prior to processing of samples for mass spectrometry was a crucial step in the protocol. The reported surface SMALPome of 3T3L1 fibroblasts consists of 205 integral membrane proteins. It is apparent that the detergent extraction method used is, in general, quantitatively more efficient at extracting proteins from the plasma membrane than SMA extraction. However, samples prepared following detergent extraction contained a greater proportion of proteins that were considered to be “non-specific” than in samples prepared from SMA extracts. Tantalizingly, it was also observed that proteins detected uniquely or highly preferentially in pull-downs from SMA extracts were primarily multi-spanning membrane proteins. These observations hint at qualitative differences between SMA and detergent extraction that are worthy of further investigation.

## 1 Introduction

Integral membrane proteins carry out a wide range of important biological functions in cells. In eukaryotic cells, integral membrane proteins are present in the intracellular organellar membranes of the endomembrane system, mitochondria, and plastids (in plants) as well as the plasma membrane. At the plasma membrane, their functions include signaling, transport, and cell adhesion. In order to investigate the structure and function of integral membrane proteins, it is often desirable to obtain purified protein. The first step in any purification of membrane proteins is their extraction from the membrane, whether they are recombinantly expressed or extracted from their natural source. Detergents, which solubilize membrane bilayers into detergent/lipid micelles and prevent hydrophobic transmembrane domains of integral membrane proteins from aggregating, have been extensively used for this purpose (Seddon et al., 2004). A disadvantage of using detergents for extraction is that integral proteins are often unstable and/or difficult to functionally characterize when removed from their natural bilayer environment (Lee et al., 2016a). Although these issues may sometimes be overcome by screening for the “correct detergent” or reconstitution of protein back into model bilayers following purification, they still pose a major bottleneck in the study of integral membrane proteins (Linke, 2009; Moraes et al., 2014).

In the past decade, an alternative to detergent has been developed for the extraction of proteins from membranes. It utilizes styrene maleic acid (SMA) copolymers to extract integral membrane proteins directly from the lipid bilayer, into nanodiscs with diameters ranging from approximately 10 to 30 nm, together with annular lipids (Jamshad et al., 2015b; Cuevas Arenas et al., 2016; Ravula et al., 2017; Overduin et al., 2021). Mechanistically, it has been proposed that SMA is aggregated in solution, disaggregating upon contact with membranes where hydrophobic styrenes insert into the core of the membrane leading to fracture and nanodisc formation (Bjornestad et al., 2021). SMA extraction entirely negates the use of detergents while retaining proteins in their surrounding lipidic environment. It is proposed that protein function is maintained within these nanodiscs. Indeed, there have been a small number of studies demonstrating that proteins such as mammalian GPCRs, extracted in nanodiscs, are in a functional conformation (Jamshad et al., 2015a; Logez et al., 2016; Wheatley et al., 2016). Unfortunately, SMA does have some limitations, such as sensitivity to divalent cations, which cause the copolymer to lose its negative charge and therefore dissociate from the nanodisc structure.

Proteins extracted by SMA or other copolymer variants are amenable to analysis by a variety of biophysical and biochemical techniques, such as small-angle scattering (SAS) techniques, CryoEM, nuclear magnetic resonance spectroscopy (NMR), native PAGE, dynamic light scattering (DLS), and circular dichroism (CD) (Farrelly et al., 2021). Mass spectrometry (MS) is another technique, which is starting to be used to characterize the strengths of polymer nanodiscs. However, “omics” studies have tended to focus on the lipid composition within nanodiscs (Barniol-Xicota and Verhelst, 2018; van 'T Klooster et al., 2020; Bada Juarez et al., 2021). There is one previous study that employs proteomics on bacterial membranes extracted with SMA (Carlson et al., 2019).

In this study, the focus is on mammalian proteins incorporated into SMALPs. We have coined the term SMALPome to mean the proteome that can be detected after extraction in SMALPs. By combining cell surface biotinylation, SMA extraction, purification of biotinylated proteins, and proteomics, we have explored the surface SMALPome of mammalian cells. As well as developing protocols for proteomics on SMA extracted proteins, the aim of the study was to compare the surface SMALPome with the surface proteome detected following detergent extraction. This would allow assessment on whether there is preferential or differential extraction of proteins with particular properties using either of the methods.

As far as we are aware, this is the first study to combine SMA extraction with a global proteomics approach in mammalian cells. We highlight some of the problems we have encountered during development of the approach and how these have been minimized. We provide evidence that there are quantitative and qualitative differences in the plasma membrane proteins detected following extraction with SMA compared with detergent.

## 2 Materials and Methods

### 2.1 Materials

SMA 2000 (a copolymer of styrene and maleic anhydride) was supplied by Cray Valley and hydrolyzed to form SMA using the protocol described by Hall et al. (2018). NC Nitrocellulose Membranes (#15249794, Cytiva Amersham™ Protran™), Gibco™ 10× concentrated Dulbecco’s phosphate-buffered saline (#14200–067), Halt™ Protease Single-Use Inhibitor Cocktail (100x) (#78430), Pierce™ Cell Surface Protein Isolation Kit (#89881), and Streptavidin-HRP (#10015714) were supplied by ThermoFisher Scientific. All other chemical reagents, cell culture media, and supplements were from Sigma Aldrich.

### 2.2 Cell Culture

3T3-L1 mouse fibroblasts were cultured in Dulbecco’s modified Eagle’s medium—high glucose [DMEM, supplemented with 10% newborn bovine serum, 2 mM glutamine, and 100 U/ml of penicillin/100 µg/ml of streptomycin (Frost and Lane, 1985)] and grown to ∼70%–80% confluency before splitting. Fibroblasts were grown to ∼90% confluency in 10-cm cell culture dishes (approximately 10^7^ cells) for experimentation.

### 2.3 Surface Biotinylation

**FIGURE 1:**
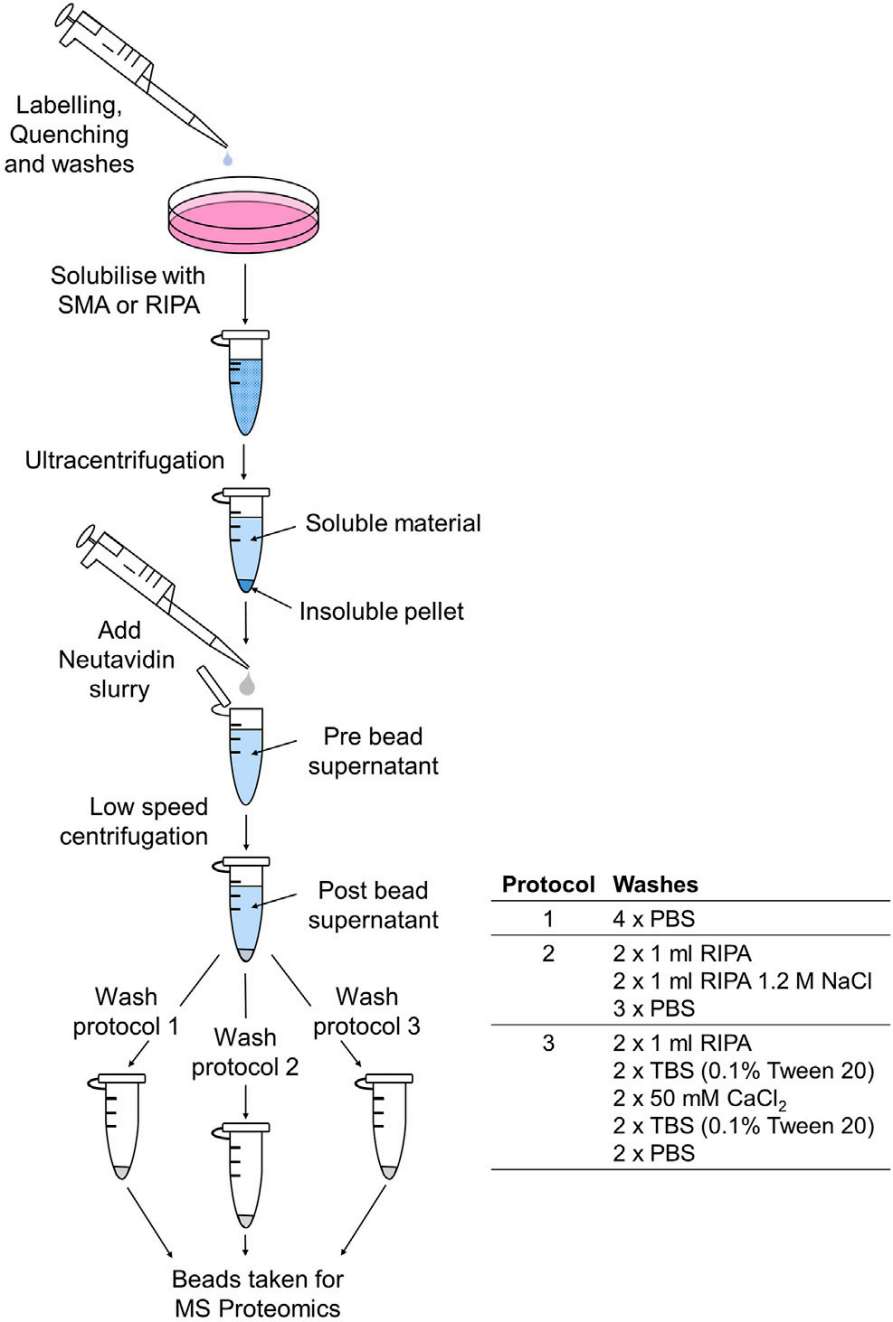
Schematic to show the biotinylation, extraction, and purification protocol for MS-proteomic analysis.

Media was removed from the fibroblasts before following the protocol from the Cell Surface Protein Isolation Kit (#89881). In summary, cells were briefly washed twice with cool PBS (12.5 mM sodium phosphate dibasic dodecahydrate, 154 mM NaCl, pH 7.4) before adding 5 ml of the prepared labeling solution, sulfo-NHS-SS-Biotin EZ-link (0.5 mg/ml in PBS) for 30 min on ice with gentle agitation to ensure even coverage (Figure 1). Quenching buffer solution was added to quench the reaction for 5 min. Cells were then briefly washed four times with 5 ml of PBS before adding extraction buffer, either 1 ml of 1.5% (w/v) SMA, 1× protease inhibitor cocktail in PBS, or 1 ml of RIPA buffer (1% Triton X-100, 0.2% SDS, 1% sodium deoxycholate in PBS), and 1× protease inhibitor cocktail for 5 min at 4°C. In some experiments, 1 ml of 3% (w/v) SMA, 1× protease inhibitor cocktail in PBS was used as the extraction buffer. Cell culture dishes were scraped with a cell scraper, and extracts were transferred to a microfuge tube. Extracts were rotated for a further 30 min at room temperature (20°C) and then centrifuged using a Beckmann Coulter TLA-100.3 fixed-angle rotor at 100,000 rpm for 30 min at 4°C. Supernatants were collected, and pellets were resuspended in 1 ml of PBS for further analysis.

### 2.4 NeutrAvidin Pull-down

Supernatants (pre-bead supernatants) were added to 100 µl of pre-washed NeutrAvidin slurry in 1.5-ml microfuge tubes and left overnight at 4°C on a rotator. Microfuge tubes were centrifuged at 100 × *g* to pellet NeutrAvidin beads. Supernatants (post-bead supernatants) were removed for further analysis. Beads were washed using three separate wash protocols. Centrifugation at 100 × *g* was required to remove the buffer at each wash step. Following the final wash, wash buffer was removed, and beads were sent for proteomics analysis. Three different wash protocols were used as follows.

### 2.5 Washing of NeutrAvidin Beads

#### 2.5.1 Wash Protocol one

Beads were washed with 4× 1 ml of PBS.

#### 2.5.2 Wash Protocol two

Beads were washed with 2× 1 ml of RIPA, 2× 1 ml of RIPA 1.2 M NaCl, and 3× PBS.

#### 2.5.3 Wash Protocol three

Beads were washed with 2× 1 ml of RIPA, 2× TBS (TBS: 0.9% NaCl, 10 mM Tris-HCl, pH 7.4; 0.1% Tween 20), 2× 50 mM CaCl_2_, 2× TBS (0.1% Tween 20), 2× PBS.

### 2.6 SDS-PAGE and Immunodetection

Samples were prepared in non-reducing SDS-sample buffer and heated for 5 min at 95°C. Samples were separated on 10% SDS polyacrylamide gels followed by transfer onto nitrocellulose using a semi-dry transfer apparatus. Blots were blocked with TBST [0.1% Tween 20 with 1% bovine serum albumin (BSA)] for at least 45 min. Streptavidin-HRP was diluted 1:10,000 in TBS, 0.2% Tween 20, 1% BSA, and added to the blot. All washes were in TBS, 0.2% Tween 20. Either GE Healthcare ECL^TM^ Select Western Blotting Detection Reagent or comparable in-house reagents were used for detection, and images were acquired with EPI Chemi II darkroom (UVP). Solubilization efficiency was measured through densitometry using Image Studio Lite software.

### 2.7 MS-Proteomics

#### 2.7.1 Sample Processing

Samples were reduced (10 mM TCEP, 55°C for 1 h), alkylated (18.75 mM iodoacetamide, room temperature for 30 min), and then digested from the beads with trypsin (2.5 µg trypsin; 37°C, overnight). The resulting peptides were desalted using a SepPak cartridge according to the instructions of the manufacturer (Waters, Milford, MA, USA). Eluate from the SepPak cartridge was evaporated to dryness and resuspended in 1% formic acid prior to analysis by nano-LC MSMS using an Ultimate 3,000 nano-LC system in line with an Orbitrap Fusion Tribrid mass spectrometer (Thermo Scientific).

#### 2.7.2 Nano-LC Mass Spectrometry

The resulting peptides were fractionated using an Ultimate 3,000 nano-LC system in line with an Orbitrap Fusion Lumos mass spectrometer (Thermo Scientific). In brief, peptides in 1% (vol/vol) formic acid were injected onto an Acclaim PepMap C18 nano-trap column (Thermo Scientific). After washing with 0.5% (vol/vol) acetonitrile 0.1% (vol/vol) formic acid peptides were resolved on a 250 mm × 75 μm Acclaim PepMap C18 reverse phase analytical column (Thermo Scientific) over a 150-min organic gradient, using seven gradient segments (1%–6% solvent B over 1 min, 6%–15% B over 58 min, 15%–32% B over 58 min, 32%–40% B over 5 min, 40%–90% B over 1 min, held at 90% B for 6 min, and then reduced to 1% B over 1 min) with a flow rate of 300 nl min^−1^. Solvent A was 0.1% formic acid and solvent B was aqueous 80% acetonitrile in 0.1% formic acid. Peptides were ionized by nano-electrospray ionization at 2.2 kV using a stainless steel emitter with an internal diameter of 30 μm (Thermo Scientific) and a capillary temperature of 300°C.

All spectra were acquired using an Orbitrap Fusion Lumos mass spectrometer controlled by Xcalibur 3.0 software (Thermo Scientific) and operated in data-dependent acquisition mode. FTMS1 spectra were collected at a resolution of 120,000 over a scan range (m/z) of 350–1,550, with an automatic gain control (AGC) target of 4 × 10^5^ and a max injection time of 50 ms. Precursors were filtered according to the charge state (to include charge states 2–7), with monoisotopic peak determination set to peptide and using an intensity threshold of 1 × 10^3^. Previously interrogated precursors were excluded using a dynamic window (40 s +/- 10 ppm). The MS2 precursors were isolated with a quadrupole isolation window of 0.7 m/z. ITMS2 spectra were collected with an AGC target of 2 × 10^4^, maximum injection time of 35 ms, and HCD collision energy of 30%.

#### 2.7.3 Data Analysis

The raw data files were processed and quantified using Proteome Discoverer software v2.1 (Thermo Scientific) and searched against the UniProt Mouse database (downloaded February 2020; 83,561 sequences) using the SEQUEST HT algorithm. Peptide precursor mass tolerance was set at 10 ppm, and MS/MS tolerance was set at 0.6 Da. Search criteria included oxidation of methionine (+15.995 Da), biotinylation of lysine (+389.6 Da), acetylation of the protein N-terminus (+42.011 Da), and methionine loss plus acetylation of the protein N-terminus (−89.03 Da) as variable modifications and carbamidomethylation of cysteine (+57.021 Da) as a fixed modification. Searches were performed with full tryptic digestion, and a maximum of two missed cleavages were allowed. The reverse database search option was enabled, and all data were filtered to satisfy false discovery rate (FDR) of 5%.

The outputs from the Proteome Discoverer were filtered to identify transmembrane proteins and proteins that have a signal sequence. First, any non-mouse contaminants were removed (i.e., contaminants = TRUE) from the data sets. Next, proteins with ≤1 unique peptide were removed from the data sets. The filtered data sets were compared with mouse proteins (86,521 proteins) in the Uniprot database identified by the following searches:

To identify integral membrane proteins: Organism [OS], Mus musculus (mouse) AND Keyword [KW] Transmembrane helix (18,359 proteins, downloaded December 2020).

To identify proteins with a signal sequence but not a transmembrane domain: Organism [OS] Mus musculus (mouse) AND PTM Processing > molecule processing. signal peptide, NOT Keyword [KW] transmembrane helix (6,683 proteins, downloaded April 2021).

To identify GPI anchor proteins associated with lipid rafts: keyword:“GPI-anchor [KW-0336]” AND organism:“Mus musculus (Mouse) [10,090]” (226 proteins, downloaded July 2021).

To identify mitochondrial transmembrane proteins: keyword:“Transmembrane [KW-0812]” keyword:“Mitochondrion [KW-0496]” AND organism:“Mus musculus (Mouse) [10,090]” (810 proteins, downloaded June 2021).

Uniprot annotation was used to determine whether a membrane protein was a single or multi-spanning transmembrane protein, using the Accession number. For those proteins where Uniprot annotation was unclear, the number of TM-helices was predicted using TMpred from the amino acid sequence (Hofmann and Stoffel, 1993).

To determine “cellular component” enrichment, datasets, in which contaminants (i.e., non-mouse proteins) and proteins with unique peptides ≤1 were removed, underwent Gene Ontology analysis, using the PANTHER statistical enrichment test (Mi et al., 2021), for PANTHER GO-Slim Cellular components. Accession number for identified proteins and Score Sequest HT, which combines several parameters, were used as input. Cellular components were deemed statistically significant using a Fisher’s exact test (*p-*value < 0.05).

#### 2.7.4 Statistical Analysis

Data handling was conducted through R software using {dplyr} and {tidyr} packages (comparing datasets) or Microsoft Excel (for removal of contaminants and ≤1 unique peptides). Please see Supplementary Table S1 for raw data and step-by-step filters. Scatterplot and notched-box plot figures were presented using R software with the {ggplot2} package.

Results were presented as mean ± SD where indicated. Two-tailed paired t-tests were used to determine statistical significance (*p*-value < 0.05) between total protein pull-down from SMA and RIPA extracts. A minimum of three experiments were used for statistical comparisons calculated in Microsoft Excel.

Protein abundances were also statistically determined using two-tailed t-test (*p*-value < 0.001). Correlation coefficient was calculated using Pearson’s correlation coefficient in R.

## 3 Results

### 3.1 SMA is Less Efficient Than Detergent When Extracting Plasma Membrane Proteins

In order to interrogate the surface proteome that could be identified following SMA and RIPA extraction, a surface labeling approach was taken. Mammalian cells (mouse 3T3L1 fibroblasts) were labeled with a membrane impermeant reagent at 4°C, which prevents exocytosis and endocytosis. Only cell surface membrane proteins with exposed extracellular domains containing Lys residues should be labeled, providing a snapshot of proteins at the cell surface at the time of labeling. Secreted proteins that remain associated with cells at the cell surface were also expected to be biotinylated. Following extraction of the labeled cells with SMA or RIPA, soluble fractions from each of the extraction procedures were used in affinity pull-down with NeutrAvidin beads to enrich for biotinylated proteins (Figure 1).

**FIGURE 2:**
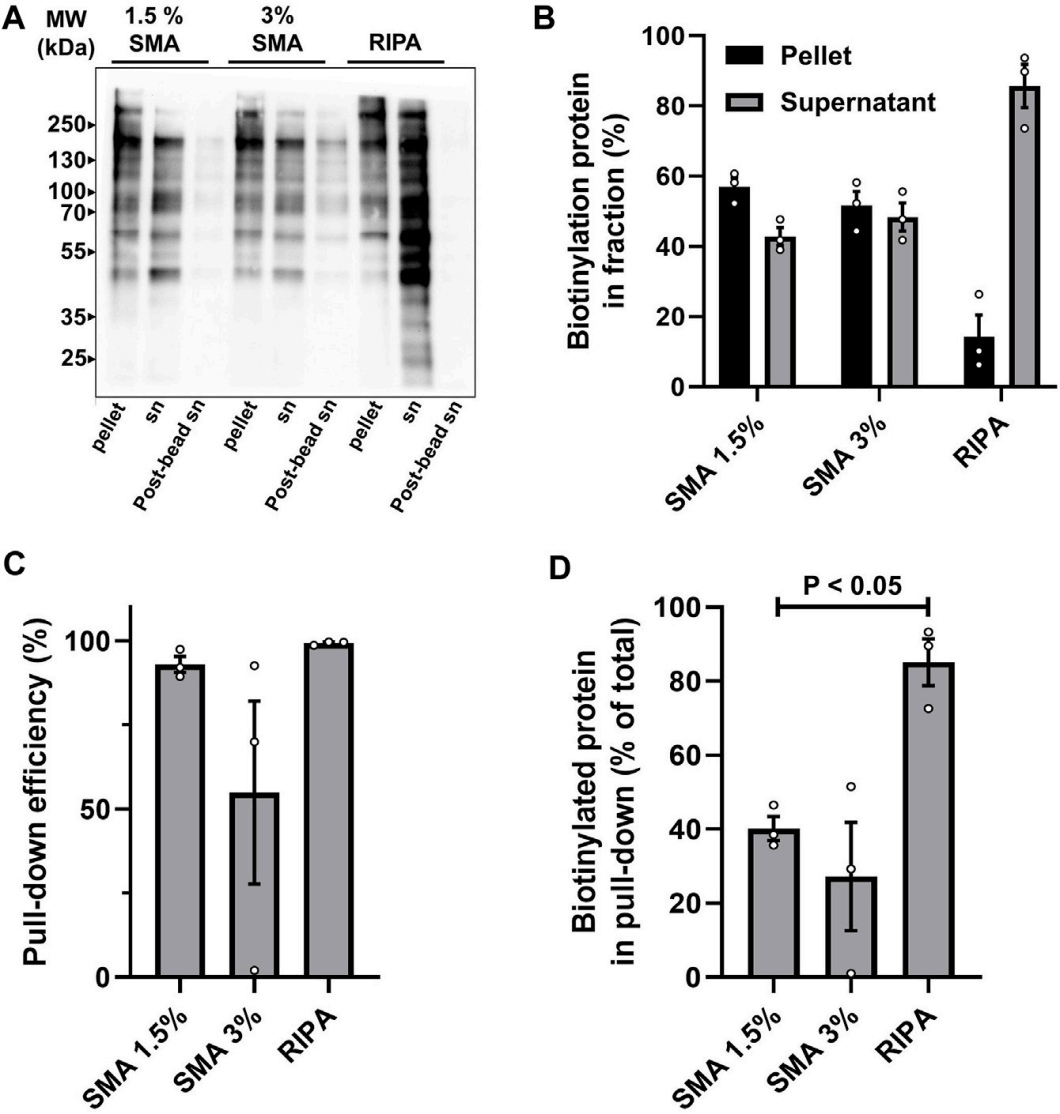
Analysis of solubilization and pull-down efficiency of surface biotinylated proteins. Cells were labeled with sulfo-NHS-SS-biotin and extracted with either 1.5% SMA, 3% SMA, or RIPA buffer. Extracts were ultracentrifuged and aliquots of pellet and supernatant (sn) fractions separated by non-reducing SDS-PAGE and transferred to nitrocellulose. NeutrAvidin pull-down was performed on supernatants and unbound material (post-bead sn) was separated on same gels. **(A)** Streptavidin-HRP detection of biotinylated proteins in pellet, supernatant, and post-bead supernatants from each of the extraction conditions. A representative blot is shown. **(B)** Quantification of solubilization efficiency. The amount of biotinylated protein in pellets and supernatants was measured by densitometry of the entire lane. The percentage of the total biotinylated protein (i.e., combined sn + pellet) in sn and pellet for each of the extraction conditions was calculated. Quantification from three independent experiments is shown (values are mean ± SD). **(C)** Quantification of pull-down efficiency from supernatants. Pull-down efficiency was determined by densitometry values of pre-bead and post-bead supernatants for each of the extraction conditions. The assumption was made that the biotinylated proteins not in the post-bead supernatants were bead associated. Quantification from three independent experiments is shown (values are mean ± SD). **(D)** Quantification of recovery of biotinylated protein on beads. Calculated by combining the solubilization efficiency and pull-down efficiency. Biotinylated protein associated with beads is expressed as percentage of total biotinylated protein in sample (i.e., combined sn + pellet) for each of the extraction conditions. Quantification from three independent experiments shows a significant difference *p* < 0.05 (indicated by bar) between the amount of biotinylated protein recovered following 1.5% SMA and RIPA extraction (values are mean ± SD, two-tailed *t*-test, *p*-value = 0.016). Original blots used for quantification are presented in Supplementary Figure S1.

Initially the solubilization efficiency of surface biotinylated proteins was compared by running samples of the soluble fractions and pellets from the different extraction procedures on non-reducing SDS-PAGE. Following transfer to nitrocellulose, biotinylated proteins were detected using Streptavidin-HRP and chemiluminescence (Figure 2). The efficiency of “pull-down” was also assessed, by comparing the levels of biotinylated proteins in the supernatants before (pre-bead supernatant) and after (post-bead supernatant) affinity precipitation using NeutrAvidin beads (Figure 2).

It is clear that extraction of surface biotinylated proteins from membranes in 1.5% (w/v) SMA, is less efficient than with RIPA, calculated at 42% and 85% in soluble fractions, respectively (Figure 2B). The efficiency of “affinity pull-down” from the supernatants was comparable, with 92% from SMA soluble fractions and 99% from RIPA soluble fractions (Figure 2C). We investigated whether increasing the concentration of SMA to 3% (w/v) in the extraction buffer could improve solubilization and pull-down efficiency. This, however, did not seem to be the case (Figure 2) and was highly variable. While the proportion of biotinylated proteins in the supernatants was, on average, marginally higher at 48% (from 42%) (Figure 2B), the efficiency of the affinity pull-down was lower, at 83% (from 92%) (Figure 2C), suggesting that the higher concentration of SMA may possibly, for unknown reasons interfere with NeutrAvidin pull-down and lead to inconsistent results. Further analysis of samples prepared following solubilization with 3% SMA was not pursued; we considered that a 92% affinity pull-down efficiency from 1.5% SMA and 99% efficiency from RIPA supernatants was acceptable. It should be noted that this equates to 40% of biotinylated proteins being recovered on NeutrAvidin beads following extraction with 1.5% SMA compared with 84% following extraction with RIPA (Figure 2D). Thus, there is a significantly lower amount (*p*-value = 0.016) of biotinylated protein recovered in pull-downs following extraction with SMA compared with RIPA.

### 3.2 Presence of Styrene Maleic Acid Co-polymer Causes Interference for MS Proteomics

To identify which proteins were present in the NeutrAvidin pull-downs from SMA and RIPA extracts, proteomic analysis was performed. The initial samples that were analyzed were from NeutrAvidin beads that had been washed in PBS (see bead wash protocol 1 in the *Methods* section). The rationale behind using PBS only washes was to preserve the structural integrity of SMA nanodiscs.

**FIGURE 3:**
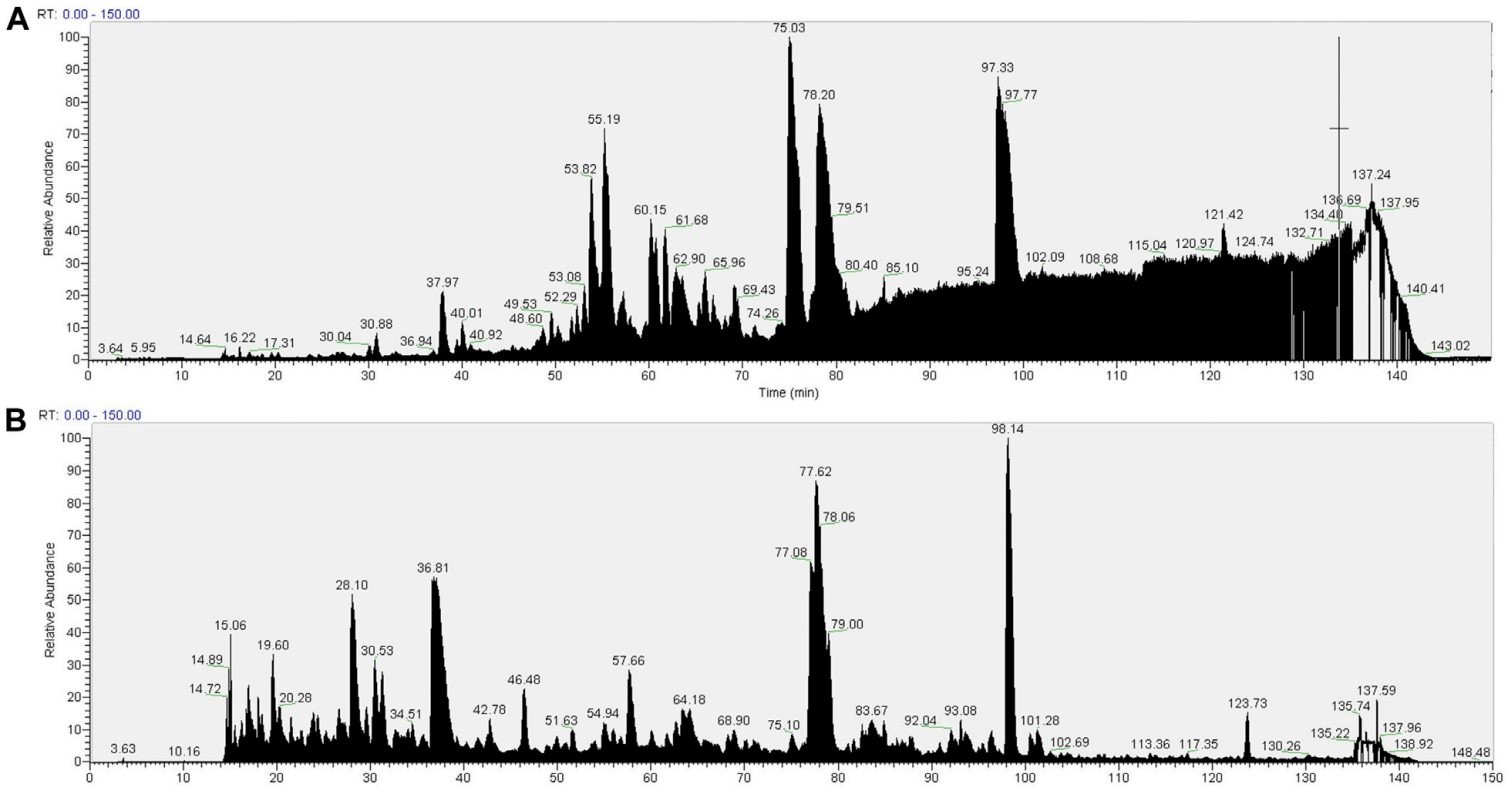
Total ion chromatograms of material associated with NeutrAvidin beads following wash procedure 1 (beads were washed with 4 × 1 ml PBS). Material associated with beads in the pull-downs from 1.5% SMA supernatants or RIPA supernatants were reduced, alkylated, and then digested from the beads with trypsin (2.5 µg trypsin; 37°C, overnight). The resulting peptides were desalted using a SepPak cartridge. Peptides were fractionated on an Acclaim PepMap C18 reverse phase analytical column over a 150-min organic gradient, using seven gradient segments. TICs for **(A)** material in pull-downs from SMA-extracted supernatants and **(B)** material in pull-downs from RIPA-extracted supernatants are shown.

The total ton chromatogram (TIC) shows a distinct interference (between about 85 and 140 min of elution) in the pull-downs prepared from cells extracted with 1.5% SMA (Figure 3A) that is not apparent in the RIPA detergent equivalent (Figure 3B). It was reasoned that the interference is caused by the presence of the SMA copolymer, as we intentionally applied a detergent-free wash protocol (wash protocol 1) to preserve the integrity of the nanodiscs.

The raw datasets of proteins identified on NeutrAvidin beads following wash protocol 1 (Supplementary Table S1, Tab A and Tab B) were filtered to remove contaminants (non-mouse proteins) and mouse proteins identified but having ≤1 unique peptides. The filtered datasets contained 903 proteins (Supplementary Table S1, Tab C) and 2,253 proteins (Supplementary Table S1, Tab D) in pull-downs from SMA and RIPA extracts, respectively (Table 1). Undoubtedly, there is a large difference in the number of proteins identified in the pull-downs from SMA soluble fractions and RIPA soluble fractions. This could be a reflection of the quantitative differences in the amount of biotinylated protein in the respective pull-downs (Figure 2) or due to the interference of the SMA copolymer with the detection of proteins by MS. It is likely to be a combination of both.

**TABLE 1:** Summary of the proteins detected following different wash protocols.

	Wash protocol 1		Wash protocol 2		Wash protocol 3	
	SMA	RIPA	SMA	RIPA	SMA	RIPA
Total proteins in raw dataset	1,670	3,149	293	2,558	737	2,158
Contaminants removed^a^	1,597	3,057	245	2,467	662	2,056
≤1 Unique peptide removed^b^	903	2,253	141	1,509	371	1,368
Proteins containing TM domain or signal peptide from the Mouse Uniprot list^c^	295	462	106	434	247	470

Numbers of proteins detected on NeutrAvidin bead pull-downs from the SMA and RIPA extracts.

a Total number of proteins left after contaminant proteins were removed from dataset (i.e. contaminants = TRUE).

b Total number of proteins remaining after removing proteins with ≤1 unique peptide from dataset (Supplementary Table S1, Tabs C, D, I, J, O and P.

c Number of proteins containing at least one transmembrane (TM) domain or a signal peptide (Supplementary Table S1, Tabs E, F, K, L, Q and R).

The expectation was that proteins associated with NeutrAvidin beads in the filtered dataset would be highly enriched for those containing at least transmembrane (TM) domain and/or a signal sequence. Proteins with these properties are likely to have non-cytoplasmic domains accessible to sulfo-NHS-SS-Biotin EZ-link. It is conceivable that some proteins will also be pulled-down indirectly by virtue of specific binding to proteins with these features. In the SMA dataset, 33% (295/903) of the proteins contained at least one predicted TM domain or a signal peptide (Table 1) (Supplementary Table S1, Tab E). In the RIPA dataset, 21% (462/2,253) of the proteins (Supplementary Table S1, Tab F) contained a predicted TM domain or a signal peptide. These figures suggested to us that wash protocol 1 was probably suboptimal and that the level of non-specific pull-down was high. Sankey plots mapping the filtering process show the number of proteins removed at each stage (Supplementary Figure S2).

### 3.3 An enhanced wash protocol reduces non-specific pull-down but does not remove SMA copolymer

The potential issues of both the presence of the SMA copolymer in the sample for proteomic analysis and non-specific pull-down of proteins was addressed by altering the NeutrAvidin bead washing procedure prior to the proteomic analysis. The biotin-NeutrAvidin interaction has extremely high affinity; therefore, there was little concern that harsher washing procedures would result in biotinylated proteins being displaced from NeutrAvidin beads. In wash protocol 2, the number of bead washes was increased and also included more stringent detergent (RIPA) and detergent with high-salt (RIPA, 1.2 M NaCl) washes. These were intended to disrupt non-specific interactions and strip away the lipid associated with copolymer/lipid/protein nanodiscs. We reasoned that detergent washes may result in disassembly of nanodiscs and removal of copolymer in addition to lipid.

**FIGURE 4:**
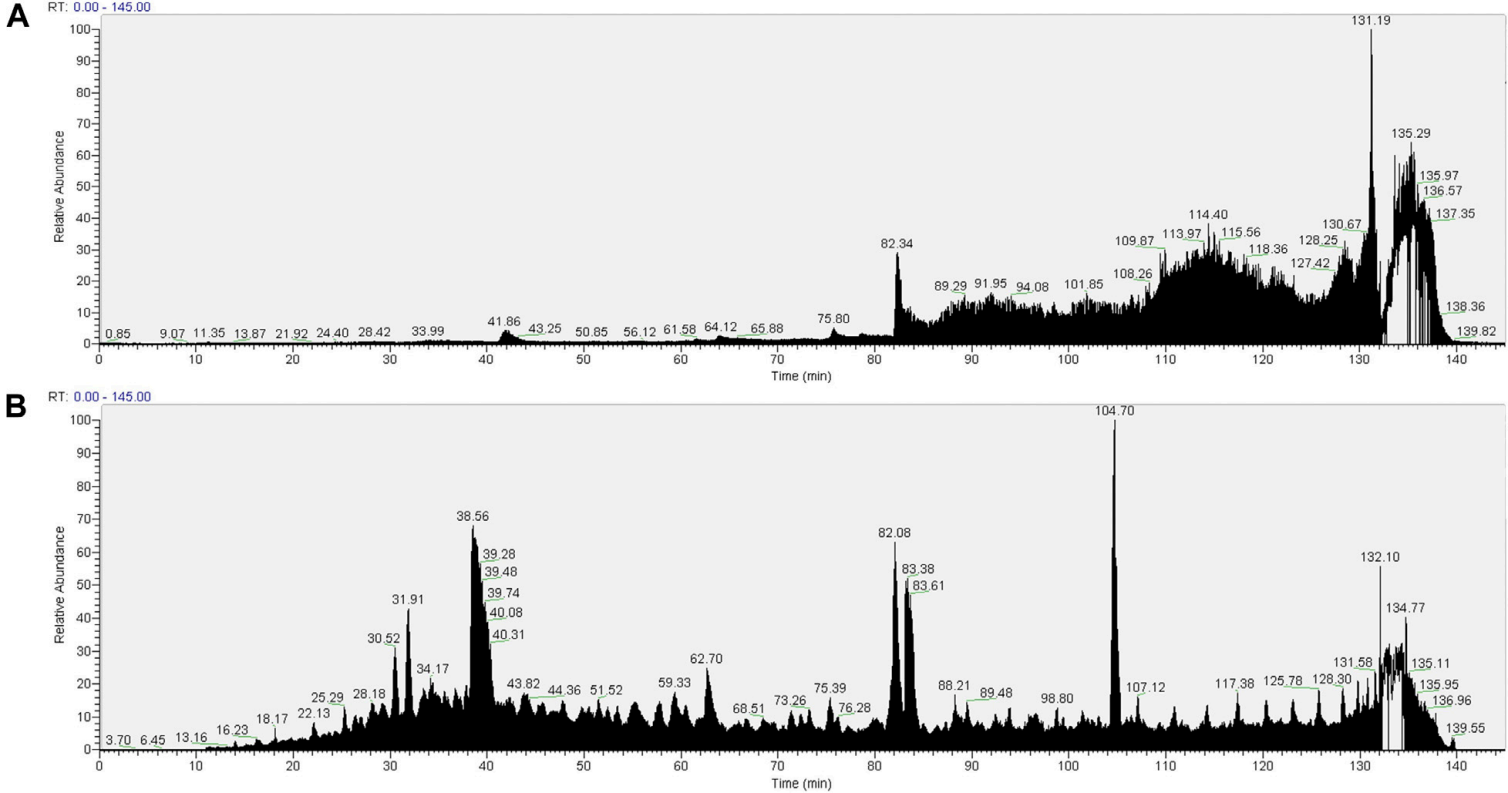
TIC of material associated with NeutrAvidin beads following wash procedure 2 (beads were washed with 2× 1 ml of RIPA, 2× 1 ml of RIPA 1.2 M NaCl, and 3× PBS). Material associated with beads in the pull-downs from 1.5% SMA supernatants or RIPA supernatants were reduced, alkylated, and then digested from the beads with trypsin (2.5 µg trypsin; 37°C, overnight). The resulting peptides were desalted using a SepPak cartridge. Peptides were fractionated on an Acclaim PepMap C18 reverse phase analytical column over a 150-min organic gradient, using seven gradient segments. TICs for **(A)** material in pull-downs from SMA-extracted supernatants and **(B)** material in pull-downs from RIPA-extracted supernatants are shown.

It is apparent from the TIC (Figure 4A) that despite the more stringent washing protocol, there is still interference of the copolymer in the pull-down from the SMA extract. This interference is not seen in the pull-down from the RIPA soluble fraction (Figure 4B). We interpret this result as wash procedure 2 still not being effective in removing the SMA copolymer from the sample.

The raw proteomic datasets were filtered (Supplementary Table S1, Tab G and Tab H) as previously. The filtered datasets contained 141 and 1,509 proteins in pull-downs from SMA and RIPA extracts, respectively (Table 1) (Supplementary Table S1, Tab I and Tab J). Of these, 75% (106/141) of proteins for SMA and 29% (434/1,509) for RIPA from the filtered datasets have a predicted transmembrane (TM) domain or a signal peptide (Supplementary Table S1, Tab K and Tab L). This indicates that wash protocol 2 dramatically reduces non-specific pull-down from the SMA extracts, while marginally reducing it in the pull-downs from the RIPA equivalent.

We concluded that the more stringent wash protocol 2 reduces non-specific protein pull-down but does not alleviate the issue of copolymer interfering with the proteomic analysis.

### 3.4 Washes That Promote Disassembly of Polymer Nanodiscs Facilitate Proteomics Analysis

Due to the ongoing presence and interference of SMA copolymer in the pull-down fractions, additional washes were introduced. It is well established that divalent cations destabilize SMA copolymer nanodiscs leading to their disassembly (Dorr et al., 2016; Lee et al., 2016b; Fiori et al., 2017; Hesketh et al., 2020). Therefore, two washes with 50 mM CaCl_2_ were included in wash protocol 3, intended to disassemble SMALPs and remove SMA from the sample.

**FIGURE 5:**
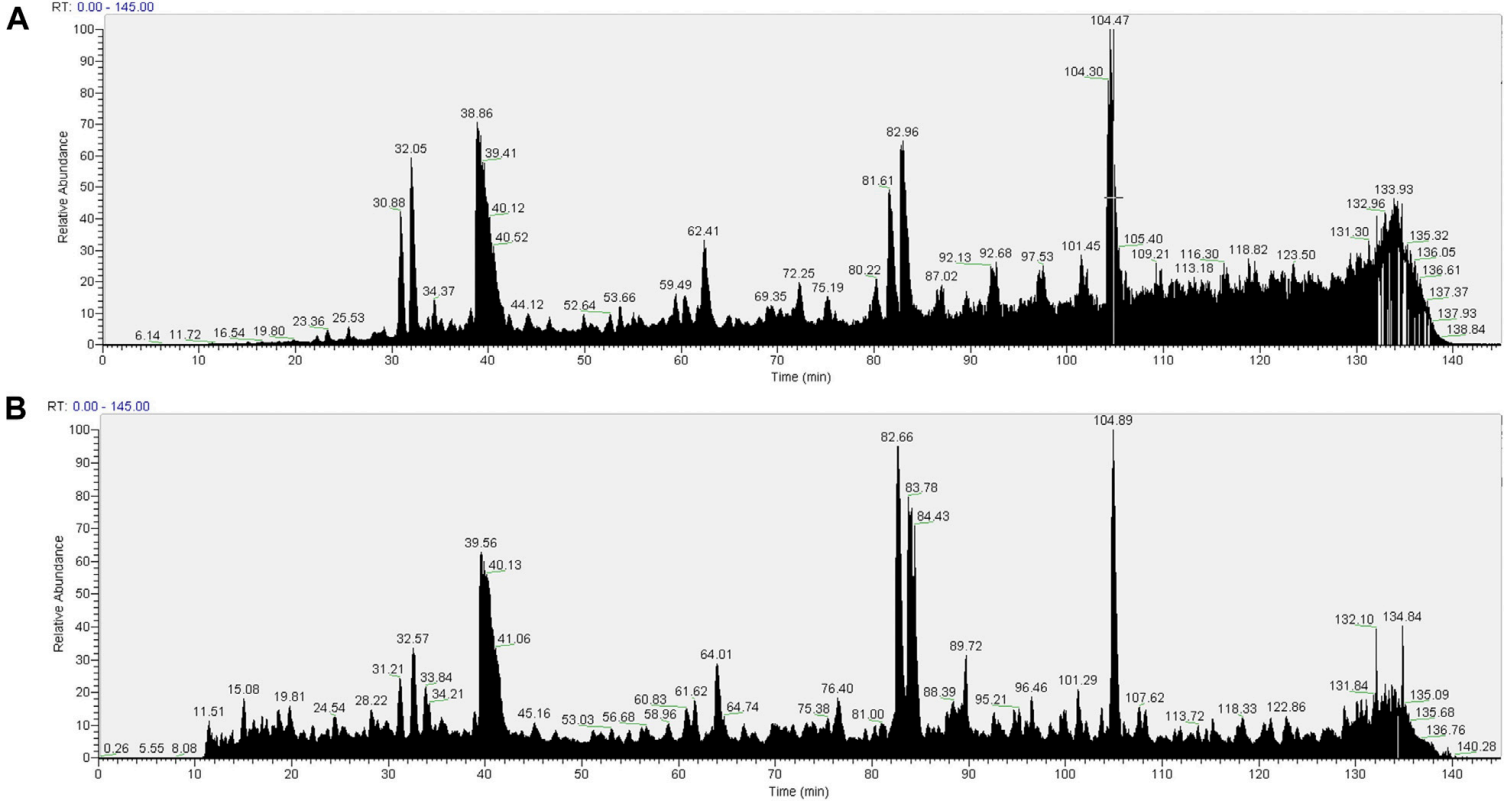
Total ion chromatograms (TICs) of material associated with NeutrAvidin beads following wash procedure 3 [beads were washed with 2× 1 ml of RIPA, 2× TBS (TBS: 0.9% NaCl, 10 mM Tris-HCl, pH 7.4; 0.1% Tween 20), 2× 50 mM CaCl2, 2× TBS (0.1% Tween 20), 2× PBS]. Material associated with beads in the pull-downs from 1.5% SMA supernatants or RIPA supernatants were reduced, alkylated, and then digested from the beads with trypsin (2.5 µg trypsin; 37°C, overnight). The resulting peptides were desalted using a SepPak cartridge. Peptides were fractionated on an Acclaim PepMap C18 reverse phase analytical column over a 150 min organic gradient, using seven gradient segments. TICs for **(A)** material in pull-downs from SMA-extracted supernatants and **(B)** material in pull-downs from RIPA-extracted supernatants are shown.

The TIC for the pull-downs from the SMA extracts (Figure 5A) show a distinct reduction in the extent of interference seen from the copolymer with previous wash protocols. This suggests that the introduction of CaCl_2_ washes was successful in removing the majority of the copolymer from the sample.

As described previously, filters were applied to the raw proteomic datasets (Supplementary Table S1, Tab M and Tab N). The filtered datasets contained 371 and 1,368 proteins in pull-downs from SMA and RIPA extracts, respectively (Table 1) (Supplementary Tables S1, Tab O and Tab P). Of these, 67% (247/371) and 34% (470/1,368) of the proteins in the filtered datasets have a predicted TM domain or a signal peptide (Supplementary Table S1, Tab Q and Tab R).

The number of transmembrane domain and signal peptide containing proteins detected in the pull-downs from SMA extracts was greatly increased to 247 (wash protocol 3) from 106 (wash protocol 2) (Table 1), indicating enhanced detection and identification of proteins by MS analysis. It seems counterintuitive that more proteins will be bound to the beads following a more stringent wash procedure; therefore, we interpret this result as the removal of copolymer facilitating identification of bound proteins that were obscured in the presence of the copolymer.

The number of proteins, containing TM domains and signal peptides, detected in the pull-downs from RIPA extracts was similar to the previous experiment, 470 (wash protocol 3) and 434 (wash protocol 2), suggesting that the addition of CaCl_2_ washes has little effect on detection by proteomics analysis of the pull-downs from RIPA extracts.

When comparing datasets from SMA and RIPA extracted samples (wash protocol 3), there was a greater number of membrane proteins annotated as being mitochondrial in the RIPA dataset, 6% (22/393) in comparison with SMA, 1% (2/205) (Supplementary Table S2).

**FIGURE 6:**
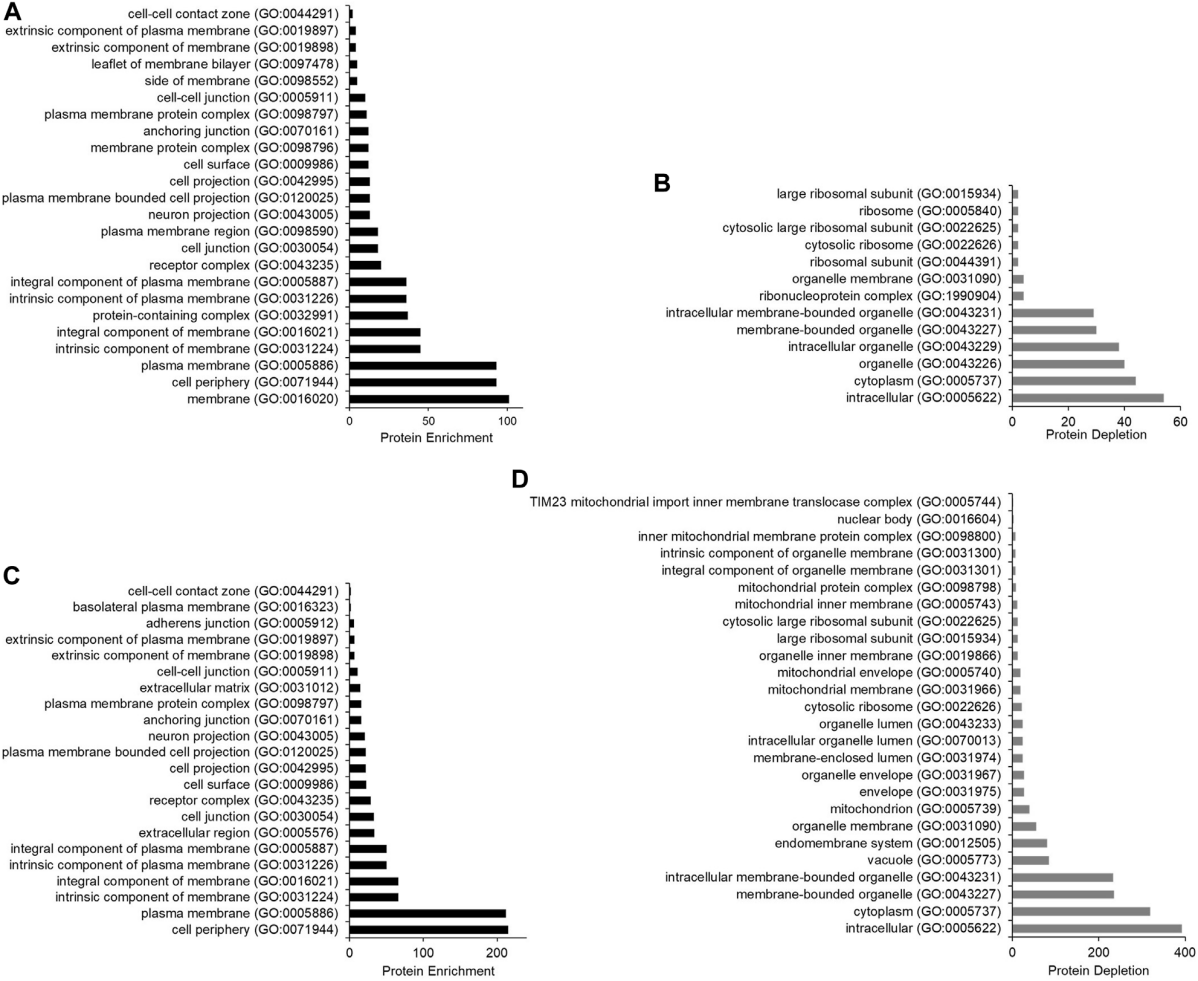
Bar chart to show the protein enrichment from samples extracted using SMA and RIPA (wash protocol 3). Gene Ontology analysis was conducted using the PANTHER statistical enrichment test (Mi et al., 2021). **(A)** protein enrichment analysis for samples extracted with SMA; **(B)** protein depletion analysis for samples extracted with SMA. **(C)** protein enrichment analysis for samples extracted with RIPA; **(D)** protein depletion analysis for samples extracted with RIPA. All cellular components underwent statistical analysis via Fisher’s exact test. Only cellular components found statistically significant are shown (*p-*value < 0.05).

Gene Ontology analysis for cellular components statistically significantly overrepresented and underrepresented in the datasets from SMA and RIPA extracted samples was performed using PANTHER (Mi et al., 2021) (Figure 6). The categories that are most highly enriched in both datasets are membrane-associated GO terms, such as cell periphery and plasma membrane (Figures 6A–C). The categories most depleted are intracellular and cytoplasm, followed by various intracellular organelles (Figures 6B–D). This confirms that with the approach we have chosen to use, cell surface biotinylation was followed by pull-down results in datasets that are highly enriched for membrane proteins and depleted of cytoplasmic and intracellular components.

### 3.5 Comparison of TM proteins in pull-downs from SMA versus RIPA soluble fractions

Filtered pull-down datasets from SMA and RIPA extracts following wash protocol 3 were used for further proteomic analysis. We consider these to be the “most complete” datasets, particularly for the pull-downs from SMA extracts, as the problems of detection due to presence of polymer were minimized.

**FIGURE 7:**
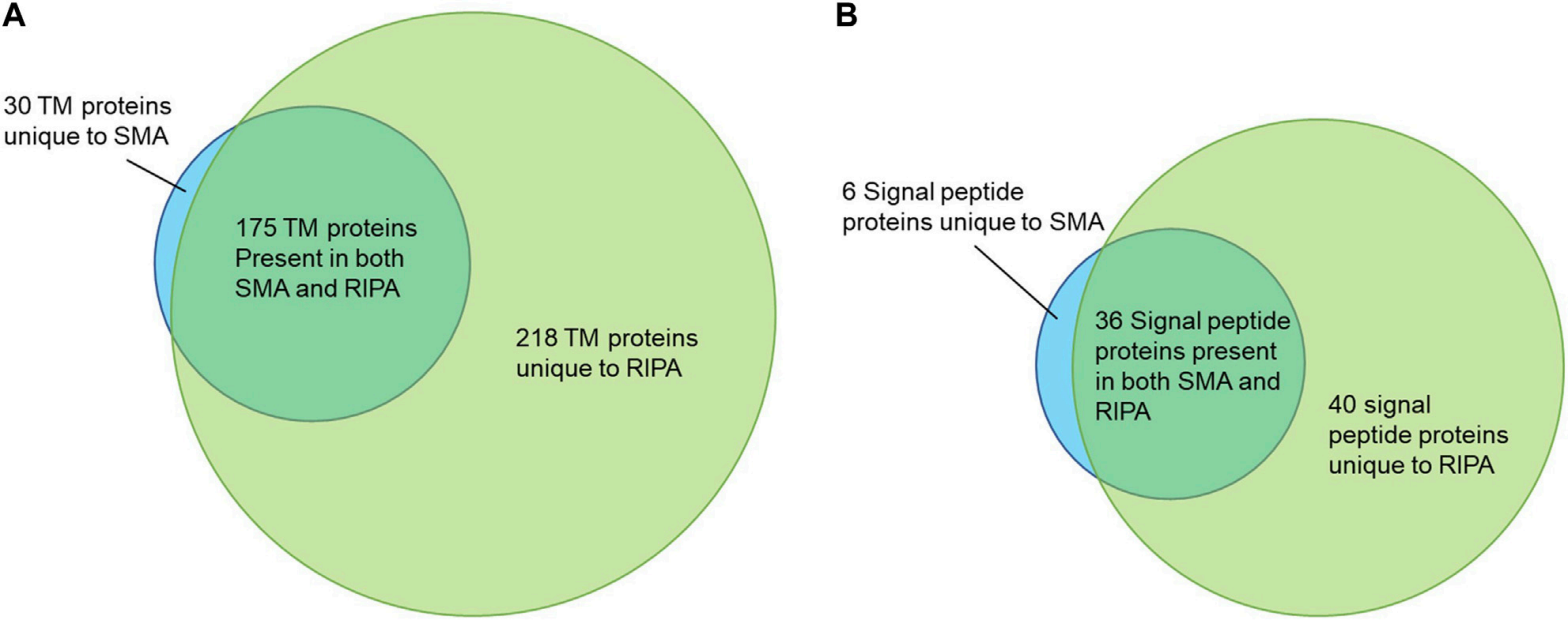
**(A)** Venn diagram showing the transmembrane (TM) proteins identified following pull-756 down from SMA and RIPA extracts. **(B)** Venn diagram showing the signal peptide containing 757 proteins without TM domains identified following pull-down from SMA and RIPA extracts.

There were 205 proteins containing at least one predicted TM domain detected in the pull-downs from the SMA and 393 from the RIPA extracts, respectively. Of these, 30 were unique to SMA and 218 unique to RIPA, with 175 TM proteins common to both datasets (Figure 7A). A Venn diagram is also shown for signal peptide containing proteins that do not contain a TM domain (Figure 7B).

**FIGURE 8:**
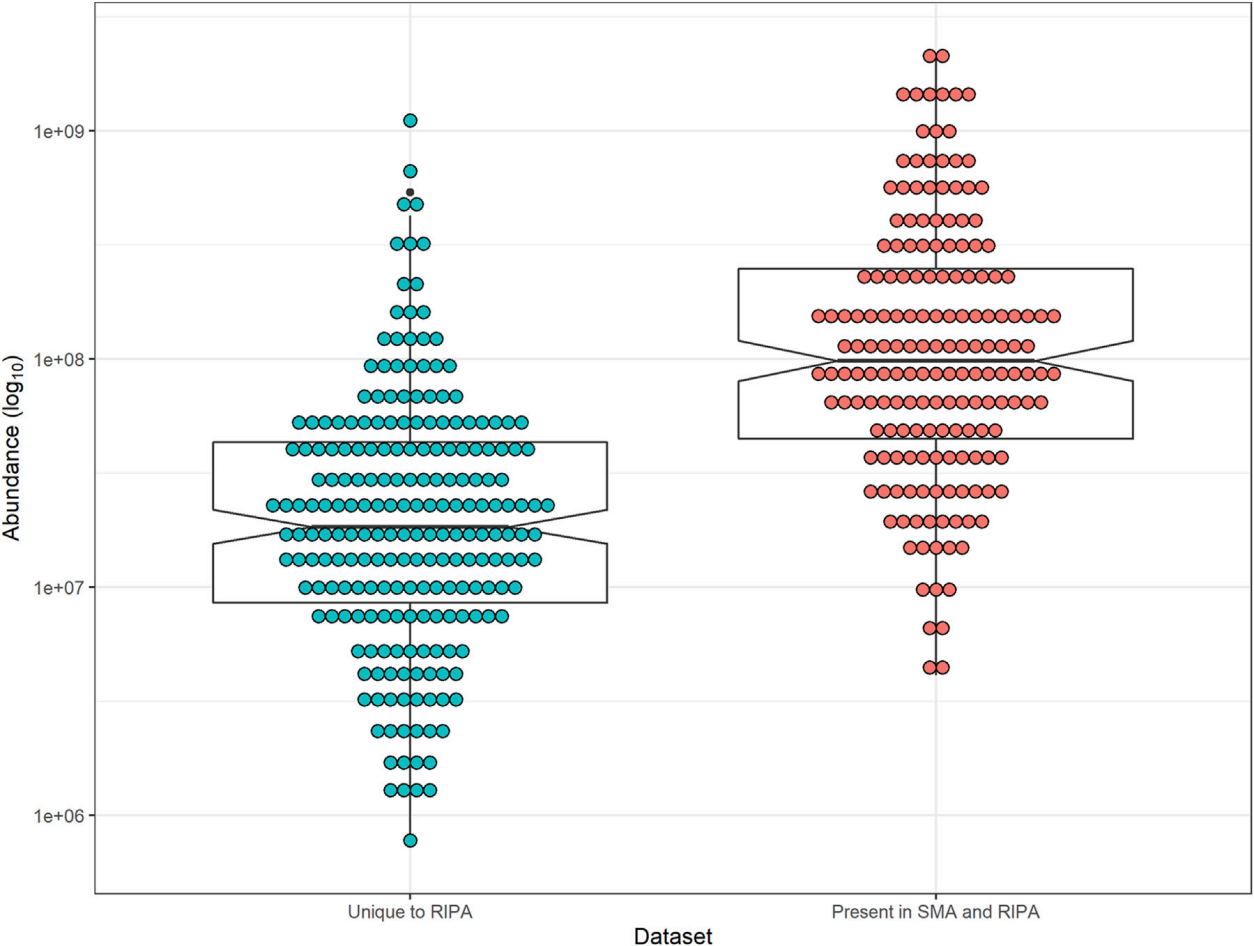
Notched-box plot showing abundance of peptides from TM proteins in pull-downs from RIPA extract. Median and interquartile range are shown. Notches indicate 95% confidence interval of median. (Blue) TM proteins unique to pull-downs from RIPA supernatants and those present in SMA (Red). Abundance (area values) were from the pull-downs from RIPA extracts (wash protocol 3, Supplementary Table S1, Tab S). Comparison between TM unique to RIPA and those in common with SMA, two-tailed *t*-test (*p*-value = 1.24 × 10^–10^).

Using the dataset generated (Supplementary Table S1, Tab S) from the pull-downs from RIPA extract (wash protocol 3), the abundances of each TM protein was plotted. The area value used for this analysis gives the average area of the three unique peptides with the largest peak area for each protein. The TM proteins were split into two groups: Those that were unique to pull-downs from RIPA extracts and those that were common between pull-downs from SMA and RIPA extracts (Figure 8).

The most abundant proteins in the pull-down from RIPA extracts are also overrepresented in the pull-downs from SMA extracts (*p*-value = 1.24 × 10^–10^) (Figure 8).

**FIGURE 9:**
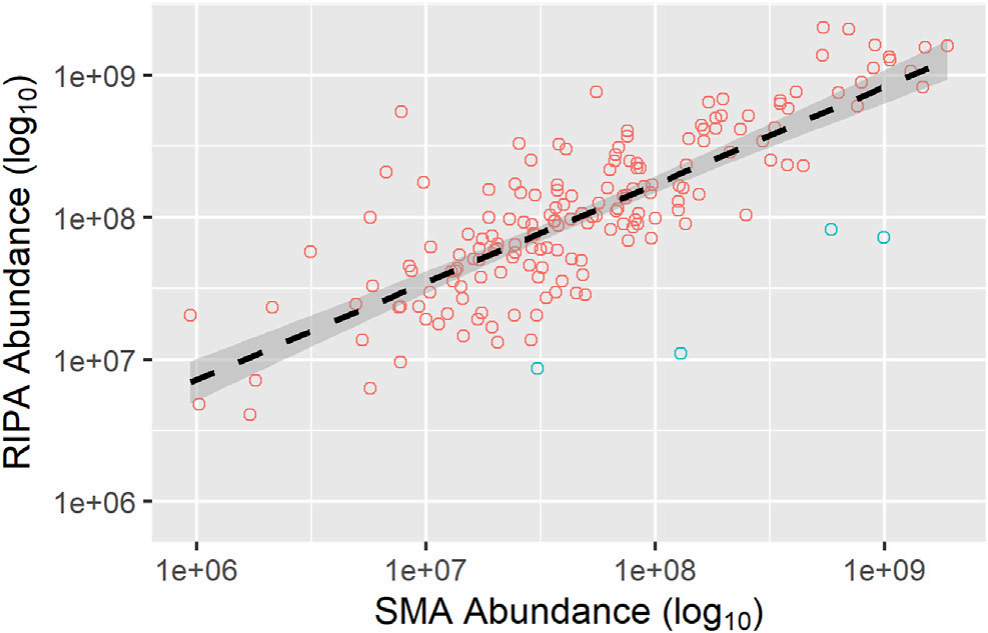
Correlation of the abundance of TM proteins common to the pull-downs from SMA and RIPA extracts. Abundances (area value) of TM proteins were taken from datasets of the pull-downs from SMA (*x*-axis) and RIPA (*y*-axis) extracts (wash protocol 3), and plotted as a scatter plot. TM proteins which have higher abundance (>3-fold) in the pull-downs from the SMA extract than from the RIPA extract are highlighted (blue circles). The regression line (black dashed) and 95% confidence interval (dark gray shaded area) are shown. Equation for regression line is y = 539.96 × 0.6876. Pearson’s correlation coefficient, r = 0.80.

Next, a correlation of the abundance of TM proteins common to both the pull-downs from the RIPA and SMA extracts was made (Figure 9). For this, a comparison was made between the dataset generated from the pull-down from the RIPA extract and that from the SMA extract (both wash protocol 3).

The abundances of the shared integral membrane proteins detected in the pull-downs from the RIPA extracts compared with the SMA extract were highly correlated (r = 0.80).

In general, the most abundant proteins in the pull-down from RIPA extract are also found in the pull-down from SMA extract, whereas those less abundant are not. This cannot, however, be the whole picture as there are 30 TM proteins detected in the pull-down from the SMA extract that are not detected in the pull-down from the RIPA extract. Of these, 16 (Table 2) are not found in the proteomics data sets of the pull-downs from any of the RIPA extracts (wash protocols 1, 2, or 3). It was observed that 11 out of the 16 (69%) TM proteins unique to SMA extracts have multiple TM regions, whereas in the pull-downs from the RIPA extracts (wash protocol 3), only (135/393) 34% of the TM proteins detected had multiple TM domains. Furthermore, when proteins that were >3 times more abundant in pull-downs from the SMA extracts than the RIPA extracts (Figure 9, blue circles) were scrutinized, all four (100%) (Table 2) were multi-spanning. Overall, 15/20 (75%) of proteins highly enriched in, or unique to, the SMA extract pull-downs compared with the RIPA equivalents were multi-spanning TM proteins (Table 2).

**TABLE 2:** Membrane proteins identified as being highly enriched or unique to pull-downs from SMA extract.

Uniprot code	Membrane protein(Gene name)	No. TM domains
Q8R0I4^a^	Tm2d2	2
Q8VI59^a^	Pcnx3	13
B2RWU5^a^	Abca7	11
Q8K2Y3^a^	Eva1b	1
Q5Y5T2^a^	Zdhhc18	4
Q61469^a^	Plpp1	6
Q3TMA0^a^	Slc16a3	12
Q61091^a^	Fzd8	7
Q542F3^a^	Slc19a1	11
A2AW86^a^	Ly75	1
Q5U647^a^	Slc1a5	9
Q6PIX5^a^	Rhbdf1	7
Q8BY89	Slc44a2 Ctl2	10
Q8C8K1^a^	Ephb4	1
A0A0R4J0A9^a^	Lrp6	1
Q8CC06^a^	Itga6	1
Q3TRK9^b^	Slc16a1	10
Q02013^b^	Aqp1	6
Q8C145^b^	Slc39a6	6
G5E829^b^	Atp2b1	10

a TM proteins that are unique or highly enriched.

b In the pull-downs from the SMA extracts compared with the RIPA extracts are shown. The number of TM-domains were taken from the annotation in the Uniprot database.

We have analyzed the data for other qualitative differences between proteins unique to the pull-downs from the RIPA extracts and those common to pull-down in both the SMA and RIPA extracts. For example, a comparison was made of the average TM domain length of all the single-spanning TM proteins in each of the groups. For proteins unique to pull-downs from the RIPA extracts, the average TM domain length is 21 residues, (wash protocol 3, n = 124 single-span TM proteins). For proteins common to pull-downs from the SMA and RIPA extracts, the average TM domain length is 21.3 residues (wash protocol 3, n = 133 single-span TM proteins). For proteins unique to the SMA extracts, the average TM domain length was 21.8 residues (n = 14 single-span TM proteins).

Of the proteins with TM domains or signal peptides, 10/247 (4%) and 15/470 (3.2%) of proteins in the pull-downs from the SMA and RIPA extracts, respectively, are annotated as having glycophosphatidylinositol (GPI) anchors in the Uniprot database (Supplementary Table S3).

## 4 Discussion

This study is the first that takes a global proteomic approach to investigate proteins that can be extracted from the plasma membrane of mammalian cells (mouse 3T3L1 fibroblasts) using SMA copolymer. This study identified 205 integral membrane proteins plus 42 proteins with signal peptides by proteomics following cell surface biotinylation, treatment of cells with SMA, removal of insoluble material, and NeutrAvidin pull-down. We consider these proteins (Supplementary Table S1, Tab R) to be the core plasma membrane SMALPome. We have made the assumption that the integral membrane proteins in the SMA soluble fractions are incorporated into nanodiscs; otherwise, they would be in the insoluble pellet fraction following ultracentrifugation. It cannot be ruled out that some of the TM proteins in this dataset are identified following non-specific pull-down, although the evidence suggests that the dataset is highly enriched for proteins with features compatible with surface biotinylation. This is further supported by PANTHER analysis, which shows cellular components associated with membranes to be enriched (Figure 6). Indeed, the enrichment following pull-down from the SMA extract (67% proteins with TM domain or signal peptide) is much higher than in the pull-downs from the RIPA extract (34% proteins with TM domain or signal peptide) despite both samples having the same wash procedure. This suggests to us that purification following SMA extraction may be more specific. This is further supported by a greater proportion of mitochondrial membrane proteins, which are almost certainly contaminants being present in pull-downs from the RIPA extracts (6%) compared with the SMA extracts (1%) (Supplementary Table S2). However, it should be re-emphasized that not all of the proteins in the datasets that are not accessible to be biotinylated should be classed as contaminants. Many may be pulled down due to specific interactions with biotinylated proteins as part of a complex. While we have not performed analysis on proteins in the datasets that are not predicted to be labeled specifically with membrane impermeant reagent, there are definitely proteins that fit this category in the datasets. An example is moesin, which is a cytoplasmic protein that interacts with the cytoplasmic domain of the integral membrane protein VCAM-1 (Barreiro et al., 2002). Moesin and VCAM-1 are detected in the pull-downs from the RIPA and SMA extracts, so we speculate that moesin is pulled down due to an association with biotinylated VCAM-1. The VCAM-1 and moesin interaction is presumably robust enough to survive the wash protocol 3 that disrupts nanodisc integrity. It still remains to be fully investigated whether protein–protein interactions are generally preserved in SMA copolymer nanodiscs. A previous study indicates that extraction with SMA may actually be disruptive to protein–protein interactions and result in protein complex disassembly (Carlson et al., 2019). This observation, if confirmed, may limit the applicability of SMA extracted samples in co-immunoprecipitation experiments.

It is almost certainly the case that the SMALPome list is incomplete for a number of reasons. First, it is apparent that a major factor in determining whether a protein is detected in the pull-downs from the SMA extract is its abundance. Due to the solubilization efficiency of the plasma membrane by SMA being lower than that with RIPA, the quantity of biotinylated proteins in pull-downs from SMA extracts are lower than from RIPA extracts (Figure 2). Other biological membranes, such as cyanobacterial thylakoid membranes are also more resistant to SMA solubilization in comparison with detergents (Brady et al., 2019). The most abundant proteins in the pull-downs from the RIPA extracts are the proteins over-represented in the pull-downs from the SMA extracts. Proteins detected in pull-downs from the RIPA extracts but not in the SMA extracts are not necessarily absent from the SMA extract pull-downs but may be below detection limit. Thus, by simply preparing more samples, it is likely that less abundant proteins will also be detected in pull-downs from the SMA extracts. Second, it should be noted that surface biotinylation requires the membrane impermeant reagent to react with lysine residues on extracellular domains of proteins. Some integral membrane proteins at the plasma membrane may not be biotinylated due to lack of sulfo-NHS-SS-Biotin EZ accessible lysine residues in extracellular domains. Indeed, we found that a majority [64% (271/423)] of membrane proteins in both datasets (wash protocol 3) were single span membrane proteins, most with large predicted extracellular domains.

An important observation was that the presence of SMA copolymer interferes with the proteomic analysis, potentially due to its heterogeneity. The SMA 2000 batch used in this study had a 2:1 ratio of styrene to maleic acid with an average M.W. of 7,200 and polydispersity of 1.8. Alternatively, the protocols used to prepare proteins for proteomics, that involve exposure to low pH, may lead to copolymer aggregation. Whatever the explanation for the interference, evidence is provided that it is optimal to use conditions that promote nanodisc disassembly (Dorr et al., 2016; Lee et al., 2016b; Fiori et al., 2017; Hesketh et al., 2020) and remove the copolymer prior to proteomic analysis. The previous study employing proteomics on SMA extracted samples of bacterial membranes used acetone precipitation of protein prior to trypsin digestion, which may be another method of removing SMA from samples (Carlson et al., 2019). It may, however, be possible to perform proteomics without removal of copolymer if extraction from membranes is with RAFT-synthesized copolymers (Craig et al., 2016; Ravula et al., 2017; Hall et al., 2018; Harding et al., 2019; Cunningham et al., 2020) or copolymers with desired properties, such as acid compatibility (Hall et al., 2018), that display much less heterogeneity or are less prone to aggregation. Such copolymers may not mask the signals from proteins so extensively and may themselves produce more discrete mass signals than the SMA copolymer used in this study.

Now that the methods for detecting membrane proteins following SMA extraction have been developed, the next step will be to perform quantitative proteomics utilizing a tandem mass tag (TMT) approach. This will allow a more comprehensive quantitative comparison of relative abundances of surface biotinylated membrane proteins extracted by SMA or RIPA (detergent). By performing extractions and pull-downs in at least triplicate experiments, proteins that show statistically validated subtle differences in abundance will be identified. This has the potential to reveal properties of membrane proteins, which facilitate preferential extraction with SMA or RIPA. It has been previously shown that size and packing density of protein complexes can influence SMA solubilization efficiencies (Swainsbury et al., 2017). A systematic analysis of proteins extracted over a range of variables such as temperature, polymer concentration, cell type, and with different polymers will be desirable to inform about the relative merits and limitations of extraction procedures. While some analysis has been carried out on the data obtained in this study, we need to be cautious in overinterpretation of observations that are not statistically validated. One such observation is that multi-spanning membrane proteins are preferentially detected in the pull-downs from the SMA extracts that are absent, or much depleted in pull-downs from the RIPA extracts. In our opinion, it is unlikely to be a chance occurrence, as having 4/4 (100%) of proteins having multiple TM domains enriched >3-fold in the pull-downs from SMA, compared with the RIPA extracts that has a probability of approximately 1 in 600 (Figure 9). This is intriguing, but currently, we have no explanation for this. It could be that proteins with multiple TM domains are extracted from membranes by SMA more efficiently than with detergent. Alternatively, pull-down of biotinylated multi-spanning membrane proteins may be hampered by, for example, steric hindrance, in RIPA extracts compared with SMA extracts.

Previous studies on model membranes have shown that lipid packing properties influence the solubilization kinetics and efficiency of SMA copolymers (Scheidelaar et al., 2015). It has also been demonstrated that SMA can selectively solubilize lipids in more fluid liquid disordered (L_d_) phase over those in more rigid gel or liquid ordered (L_o_) phase (Dominguez Pardo et al., 2017). Thus, it has been suggested that “SMA resistance” rather than detergent resistance may be an attractive approach for the isolation of ordered domains from biological membranes (Dominguez Pardo et al., 2017). The “classical” ordered domains, lipid rafts were first hypothesized in the late 1980s (Simons and Van Meer, 1988). One of the most widely used, but also most controversial, techniques for studying lipid rafts is resistance to “cold detergent extraction” (Munro, 2003). Our observation that only 42% of surface biotinylated proteins were extracted by SMA from the plasma membrane hints that it is more selective than detergent. However, the proteomic analysis did not reveal evidence that SMA extraction is superior to detergent extraction in distinguishing between L_o_ and L_d_ domains of biological membranes. GPI anchored proteins, commonly used as lipid raft markers (Levental et al., 2010), are not relatively depleted in pull-downs from the SMA compared with RIPA extracts. A deficit in GPI anchored proteins might be expected if SMA is more selective than detergent in solubilizing L_o_ over L_d_ domains of membranes. Furthermore, there was no difference in TM domain length between single spanning TM proteins unique to pull-down following RIPA extraction compared with those common to pull-downs from both extraction methods. As lipid rafts are proposed to be “thicker” than non-raft domains, it might be expected that proteins unique to RIPA extraction would have longer TM domains (Lorent et al., 2017) if detergent is less selective than SMA in solubilizing different lipid phases. It should be noted that we extracted with both RIPA and SMA at 20°C, which will result in lipid phases in cellular membranes being “non-physiological.” Possibly, more membrane would be in the L_o_ phase at 20°C than at the physiological temperature of 37°C. Artifactual phase separation has been a major critique of the “cold detergent extraction” method and is also a valid criticism of our study (Munro, 2003). Extraction at 37°C, combined with quantitative proteomics, may be needed to fully assess the utility of SMA extraction over detergent extraction in the study of membrane domain architecture in mammalian cells.

In conclusion, this study has seen the development of methodology for performing SMALPomics, large-scale proteomics on proteins that have been extracted from membranes in nanodiscs. To our knowledge, this is the first study to report an unbiased proteomic analysis of mammalian plasma membrane proteins extracted with copolymer. Our data indicate that there are quantitative and qualitative differences between SMA and detergent extraction that warrant further investigation.

## Data Availability

Supporting information is freely available for download in the Bath Research Data Archive; DOI: https://doi.org/10.15125/BATH-01051. The mass spectrometry proteomics data have been deposited to the ProteomeXchange Consortium via the PRIDE (Perez-Riverol et al., 2019) partner repository with the dataset identifier PXD027735.
