# Supplementary material for: Development of Methodology to Investigate the Surface SMALPome of Mammalian Cells
Source: Front Mol Biosci. 2021 Nov 18;8:780033. doi: 10.3389/fmolb.2021.780033 (PMC8637157; doi:10.3389/fmolb.2021.780033)
Supplement: Supplementary file 4 [file DataSheet1.docx]

Supplementary Material

## Supplementary Figures


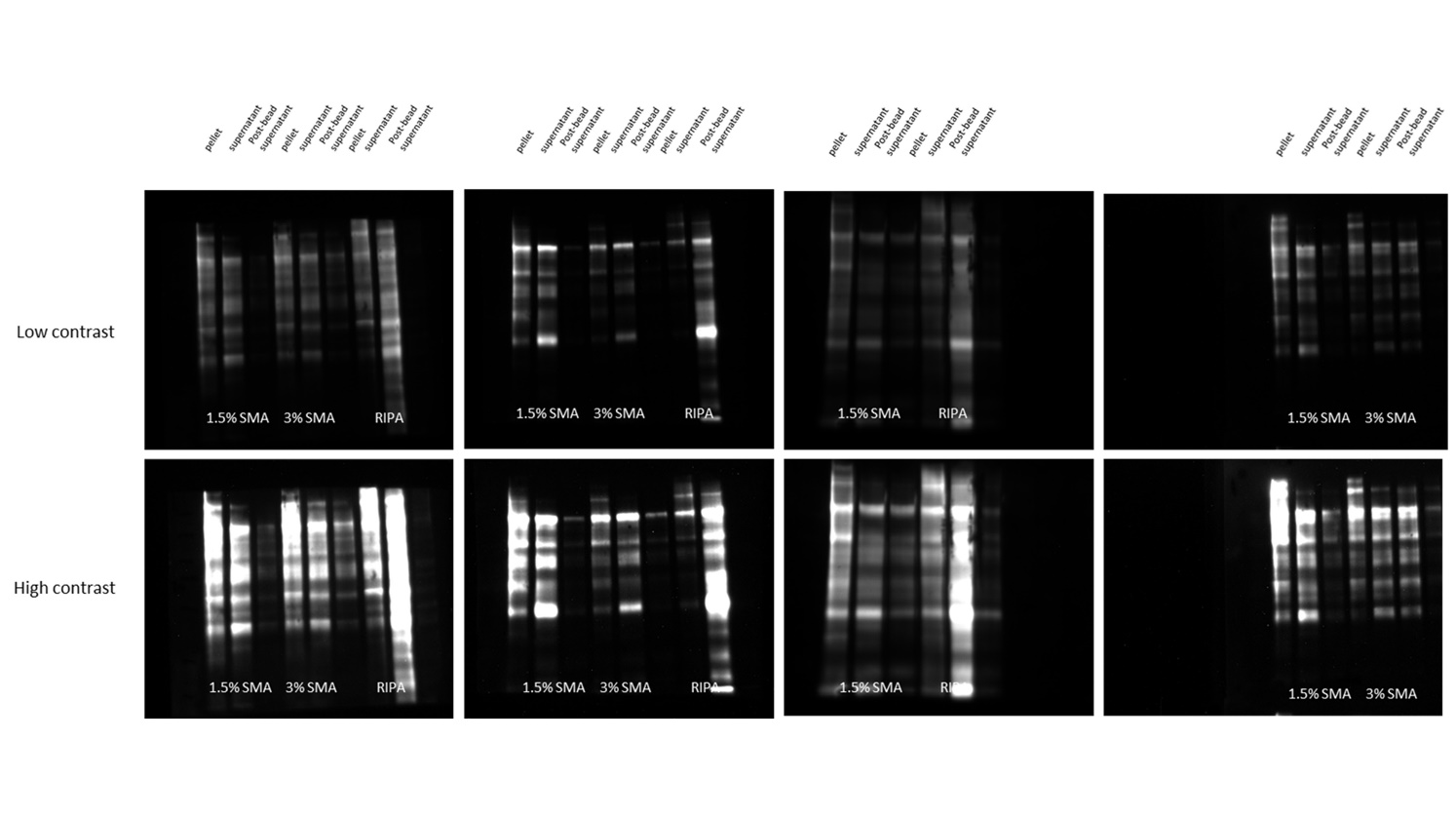


**Supplemental Figure 1.** Streptavidin-HRP detection of biotinylated proteins in pellet, supernatant, and post-bead supernatants from each of the extraction conditions. Blots used for quantification in Figure 2 are shown at two different contrasts (not inverted).


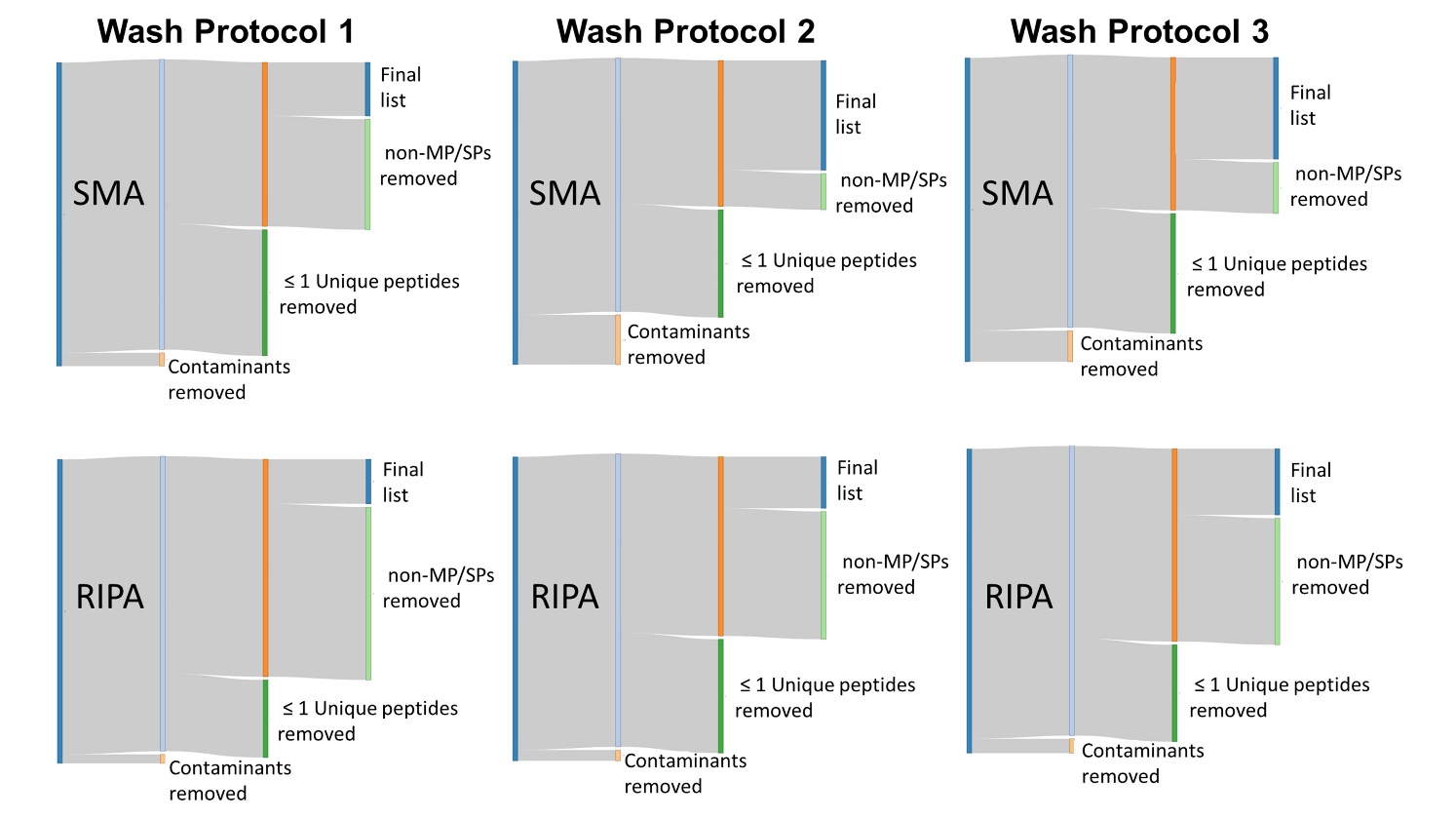


**Supplementary Figure 2.** Sankey plots mapping the filtering process to show the number of proteins removed by each filter. Comparisons between pull-downs from SMA and RIPA extracts from the three separate wash protocols (Wash protocol, 1, 2 and 3) are shown. The first filter removed non-mouse contaminants. Second filter removed proteins identified by less than 2 unique peptides. Proteins not containing a transmembrane (TM) domain or signal peptide (SP) from Mouse Uniprot list were removed leaving the final list.
